# Japanese Encephalitis Vaccine: Recommendations of the Advisory Committee on Immunization Practices

**DOI:** 10.15585/mmwr.rr6802a1

**Published:** 2019-07-19

**Authors:** Susan L. Hills, Emmanuel B. Walter, Robert L. Atmar, Marc Fischer, ACIP Japanese Encephalitis Vaccine Work Group, Emmanuel Walter, Robert L. Atmar, Elizabeth Barnett, Alan Barrett, Joseph A. Bocchini, Lin Chen, Eric Deussing, Doran Fink, Michael Holbrook, Myron Levin, Anthony Marfin, Cody Meissner, Robert Schechter, David Shlim, Mary Wilson, Marc Fischer, J. Erin Staples, Steven Waterman, Mark Gershman, Terri Hyde, Michael M. McNeil, Susan L. Hills

**Affiliations:** ^1^Division of Vector-Borne Diseases, National Center for Emerging and Zoonotic Infectious Diseases, CDC, Fort Collins, Colorado; ^2^Duke University School of Medicine, Durham, North Carolina; ^3^Baylor College of Medicine, Houston, Texas; Duke University School of Medicine, Durham, North Carolina.; Baylor College of Medicine, Houston, Texas; Boston Medical Center, Boston, Massachusetts; University of Texas, Medical Branch, Galveston, Texas; Louisiana State University, Baton Rouge, Louisiana; Mount Auburn Hospital, Cambridge,; Massachusetts; Department of Defense, Atlanta, Georgia; Food and Drug Administration, Bethesda, Maryland; Battelle Memorial Institute, Frederick, Maryland; University of Colorado, Denver, Colorado; PATH, Seattle, Washington; Tufts University, Boston, Massachusetts; California Department of Public Health, California; Jackson Hole Travel and Tropical Medicine, Jackson, Wyoming; University of California, San Francisco, California.; CDC, Fort Collins, Colorado; CDC, Fort Collins, Colorado; CDC, San Juan, Puerto Rico; CDC, Atlanta, Georgia; CDC, Atlanta, Georgia; CDC, Atlanta, Georgia.; CDC, Fort Collins, Colorado.

## Abstract

This report updates the 2010 recommendations from the CDC Advisory Committee on Immunization Practices (ACIP) regarding prevention of Japanese encephalitis (JE) among U.S. travelers and laboratory workers (Fischer M, Lindsey N, Staples JE, Hills S. Japanese encephalitis vaccines: recommendations of the Advisory Committee on Immunization Practices (ACIP). MMWR Recomm Rep 2010;59[No. RR-1]). The report summarizes the epidemiology of JE, describes the JE vaccine that is licensed and available in the United States, and provides recommendations for its use among travelers and laboratory workers.

JE virus, a mosquitoborne flavivirus, is the most common vaccine-preventable cause of encephalitis in Asia. JE occurs throughout most of Asia and parts of the western Pacific. Approximately 20%–30% of patients die, and 30%–50% of survivors have neurologic, cognitive, or behavioral sequelae. No antiviral treatment is available.

Inactivated Vero cell culture–derived JE vaccine (Ixiaro [JE-VC]) is the only JE vaccine that is licensed and available in the United States. In 2009, the U.S. Food and Drug Administration (FDA) licensed JE-VC for use in persons aged ≥17 years; in 2013, licensure was extended to include children aged ≥2 months.

Most travelers to countries where the disease is endemic are at very low risk for JE. However, some travelers are at increased risk for infection on the basis of their travel plans. Factors that increase the risk for JE virus exposure include 1) traveling for a longer period; 2) travel during the JE virus transmission season; 3) spending time in rural areas; 4) participating in extensive outdoor activities; and 5) staying in accommodations without air conditioning, screens, or bed nets. All travelers to countries where JE is endemic should be advised to take precautions to avoid mosquito bites to reduce the risk for JE and other vectorborne diseases. For some persons who might be at increased risk for JE, the vaccine can further reduce the risk for infection. The decision about whether to vaccinate should be individualized and consider the 1) risks related to the specific travel itinerary, 2) likelihood of future travel to countries where JE is endemic, 3) high morbidity and mortality of JE, 4) availability of an effective vaccine, 5) possibility (but low probability) of serious adverse events after vaccination, and 6) the traveler’s personal perception and tolerance of risk.

JE vaccine is recommended for persons moving to a JE-endemic country to take up residence, longer-term (e.g., ≥1 month) travelers to JE-endemic areas, and frequent travelers to JE-endemic areas. JE vaccine also should be considered for shorter-term (e.g., <1 month) travelers with an increased risk for JE on the basis of planned travel duration, season, location, activities, and accommodations and for travelers to JE-endemic areas who are uncertain about their specific travel duration, destinations, or activities. JE vaccine is not recommended for travelers with very low-risk itineraries, such as shorter-term travel limited to urban areas or outside of a well-defined JE virus transmission season.

## Introduction

Japanese encephalitis (JE) virus, a mosquitoborne flavivirus, is the most common vaccine-preventable cause of encephalitis in Asia (*1*,*2*). JE occurs throughout most of Asia and parts of the western Pacific (*3*,*4*). Approximately 20%–30% of patients die, and 30%–50% of survivors have neurologic, cognitive, or behavioral sequelae (*5*–*7*). In countries where the disease is endemic, JE primarily affects children. Although rare, travel-associated JE can occur among persons of any age (*8*–*10*). For most travelers to Asia, the risk for JE is very low but varies based on travel duration, season, location, activities, and accommodations (*9*,*11*).

JE virus is maintained in an enzootic cycle between mosquitoes and amplifying vertebrate hosts, primarily pigs and wading birds (*12*,*13*). JE virus is transmitted to humans by infected mosquitoes (*1*). JE virus transmission occurs primarily in rural agricultural areas. In most temperate areas of Asia, JE virus transmission is seasonal, and large outbreaks can occur. In the subtropics and tropics, transmission can occur year-round, often intensifying during the rainy season.

Inactivated Vero cell culture–derived JE vaccine (Ixiaro [JE-VC]) is the only JE vaccine that is licensed and available in the United States. An inactivated mouse brain–derived vaccine (JE-VAX [JE-MB]) has been licensed in the United States since 1992 but is no longer produced, and all remaining doses expired in 2011. In 2009, the U.S. Food and Drug Administration (FDA) licensed JE-VC for use in persons aged ≥17 years (*14*). In 2013, licensure was extended to include children aged ≥2 months. This report updates the 2010 Advisory Committee on Immunization Practices (ACIP) recommendations for use of JE vaccine among U.S. travelers and laboratory workers (*15*).

## Methods

The ACIP JE Vaccine Work Group was initially formed in 2006 to review and update information on JE vaccines available in the United States. FDA licensed JE-VC for adults aged ≥17 years in 2009. Updated ACIP recommendations for use of JE vaccine among U.S. travelers and laboratory workers were published in 2010 (*15*). ACIP subsequently approved recommendations for use of a booster dose of JE-VC in adults in 2011 and recommendations for use of JE-VC in children in 2013 after the FDA extension of JE-VC licensure to include children aged ≥2 months (*16*,*17*). The ACIP JE Vaccine Work Group was then disbanded and reformed in 2015 with revised membership. The objectives of the work group were to 1) review newly available safety and immunogenicity data for JE-VC, 2) review updated information on the epidemiology and risk for JE in travelers, and 3) review recommendations for use of JE vaccine in consideration of these data. Work group members included persons with expertise in JE, infectious diseases, pediatrics, travel medicine, public health, vaccination safety, and vaccine policy. The work group met approximately 34 times by teleconference during March 2015–January 2019. Presentations on vaccine immunogenicity and safety and on other topics related to the development of the JE vaccine recommendations were made to ACIP by the manufacturer or work group members.

Grading of Recommendations, Assessment, Development, and Evaluation (GRADE) methods were used to review and evaluate newly available data (*18*,*19*). Additional factors also were assessed in developing JE vaccine recommendations as outlined in the Evidence to Recommendations framework, including target population values, stakeholder acceptability, and feasibility of implementation (*19*,*20*). Details on the methods used for GRADE, including the search protocol, databases searched, and inclusion criteria, a summary of the evidence, the grading of the evidence, and information on the additional factors considered, are provided in *Japanese Encephalitis Vaccine Evidence to Recommendations* (*19*). The work group presented preliminary recommendations to ACIP during its October 2018 meeting. Proposed recommendations were presented to ACIP and approved at the February 2019 meeting. ACIP will review additional data as they become available, and recommendations will be updated as needed.

## Background

### JE Virus Description

JE virus, an arthropodborne virus (arbovirus), is a single-stranded RNA virus that belongs to the genus *Flavivirus* and is closely related to West Nile, St. Louis encephalitis, yellow fever, and dengue viruses (*21*,*22*). Five genotypes of JE virus have been identified (*23*). Until the 1990s, the dominant JE virus genotype in Asia was genotype III but is now genotype I (*23*).

### JE Virus Transmission

**FIGURE 1:**
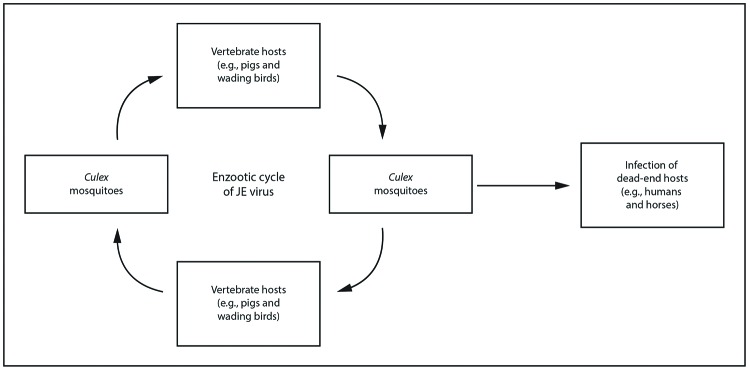
Transmission cycle of Japanese encephalitis virus* **Abbreviation:** JE = Japanese encephalitis. * JE virus is transmitted in an enzootic cycle between *Culex* mosquitoes and amplifying vertebrate hosts, primarily pigs and wading birds. Humans are a dead-end host in the JE virus transmission cycle, with brief and low levels of viremia. Humans play no role in the maintenance or amplification of JE virus, and the virus is not transmitted directly from person to person.

JE virus is transmitted in an enzootic cycle between mosquitoes and amplifying vertebrate hosts, primarily pigs and wading birds such as herons and egrets (Figure 1) (*13*,*24*–*28*). Because of rapid population turnover with numerous susceptible offspring and the development of high-titer viremia, domestic pigs are the main source of infection for mosquitoes that transmit JE virus to humans (*12*,*28*–*32*).

*Culex* mosquitoes, especially *Cx. tritaeniorhynchus,* are the principal vector for JE virus transmission in most of Asia (*12*,*13*,*24*,*27*,*33*–*40*). *Cx. tritaeniorhynchus* is an evening- and nighttime-biting mosquito that feeds preferentially on large domestic animals and birds but only infrequently on humans (*41*). *Cx. tritaeniorhynchus* feed most often in the outdoors, with peak feeding activity occurring after sunset (*41*). Larvae are found in flooded rice fields, marshes, and other stagnant collections of water (*38*,*39*). In temperate zones, the mosquito is present in the greatest density during June–November and is inactive during winter months (*12*,*26*,*42*). In certain parts of Asia and the Western Pacific, other mosquito species also are important JE virus vectors (*13*,*37*,*39*,*43*).

Infected mosquitoes transmit JE virus to humans. Humans are considered dead-end hosts in the JE virus transmission cycle because they do not develop a level or duration of viremia sufficient to infect mosquitoes (*13*,*44*). Therefore, travelers with JE virus infection who return to nonendemic areas pose minimal or no risk for subsequent transmission of the virus.

JE virus is not spread from person to person through direct contact. A small number of cases of transplacental transmission of JE virus has been reported. Four miscarriages were documented among nine infected pregnant women during outbreaks in India (*3*,*45*,*46*). All of the women who miscarried were in the first or second trimester of pregnancy, and JE virus was isolated from one of the four aborted fetuses. JE virus transmission through blood transfusion has been documented in a JE-endemic area, and on the basis of experience with similar flaviviruses, organ transplantation is considered a potential mode of transmission (*47*,*48*). In a laboratory setting, JE virus might be transmitted through accidental percutaneous exposure, or theoretically, mucosal or inhalational exposure. At least 22 laboratory-acquired JE virus infections have been reported (*49*).

### Epidemiology of JE

#### Geographic Distribution and Spread

**FIGURE 2:**
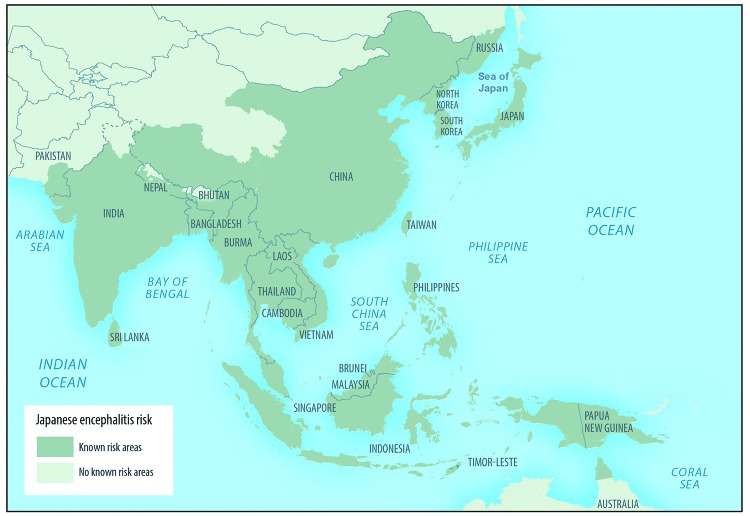
Approximate geographic range of Japanese encephalitis **Source:** Hills SL, Lindsey NP, Fischer M. Japanese encephalitis. In: CDC Yellow Book 2020: health information for international travel. New York, NY: Oxford University Press; 2019:248–57.

JE occurs throughout most of Asia and parts of the western Pacific (Figure 2). During the first half of the 20th century, the disease was recognized principally in temperate areas of Asia including Japan, Korea, Taiwan, and China (*50*–*54*). The virus then spread south and west, with increased transmission reported in Southeast Asia, India, Bangladesh, Sri Lanka, and Nepal (*36*,*54*–*68*). In the 1990s, JE virus spread east and was recognized for the first time in Saipan and then Australia, initially in the outer Torres Strait Islands and subsequently on the northern mainland (*43*,*69*,*70*). More recently, transmission also has been detected in new areas, including in Tibet and mountain districts in Nepal (*71*,*72*). The reasons for this increased geographic distribution are uncertain but might include population shifts or changes in climate, ecology, agricultural practices, animal husbandry, or migratory bird patterns (*39*,*56*,*70*). These factors could contribute to further spread, including beyond Asia and the western Pacific.

#### Incidence

In the early 1970s, approximately 100,000 cases of JE were reported each year, with the vast majority from China (*54*). Because of vaccine use, increased urbanization, changes in agricultural practices, and mosquito control, annual JE case counts have decreased substantially. Up to 5,000 cases of JE are reported to the World Health Organization (WHO) each year (*73*). However, this number likely represents an underestimate of the actual number of cases because of limited diagnostic testing and surveillance capacity in many countries with endemic JE (*5*,*7*). In 2011, taking into account the status of vaccination programs at that time, a systematic review estimated that 67,900 JE cases typically occurred annually, with an overall incidence of 1.8 cases per 100,000 population. In children aged <15 years, the incidence was estimated to be 5.4 cases per 100,000 (*5*). However, incidence can vary substantially by year and area. Before the introduction of vaccination programs, the highest risk areas in Asia had incidence rates of laboratory-confirmed JE as high as 20 cases per 100,000 children per year (*5*,*74*–*76*). In countries with vaccination programs with high coverage, JE incidence is now less than one case per 100,000 children per year (*5*,*77*).

#### Ecologic and Seasonal Patterns

The risk for JE varies by local ecology and season. JE virus transmission primarily occurs in rural agricultural areas, often associated with rice production and flood irrigation, where large numbers of vector mosquitoes breed in proximity to animal reservoirs (*24*,*27*). In some areas of Asia, these ecologic conditions might occur near, or within (although rare), urban centers (*78*–*80*).

In temperate areas of Asia (e.g., China, Japan, Nepal, northern Vietnam, northern India, South Korea, and Taiwan), JE virus transmission is seasonal, and human disease usually peaks in the summer and fall (*50*,*52*,*53*,*57*,*66*,*68*,*81*). The peak months of transmission and the length of the season vary by region, and large, explosive outbreaks can occur. In the subtropics and tropics, transmission can occur year-round, often with a peak during the rainy season (*56*,*58*,*62*,*76*,*82*).

#### Age-Specific Patterns

In areas with endemic JE, the disease primarily affects children, with the vast majority of cases occurring among children aged <15 years; most adults have protective immunity after natural exposure to the virus (*52*,*53*,*57*–*59*,*66*,*68*,*76*,*83*,*84*). However, in areas with childhood JE vaccination programs, the overall incidence of JE decreases, with a greater proportion of cases occurring among adults (*50*,*51*,*85*,*86*). Outbreaks that predominantly affected older adults have been reported in Japan, China, and India (*87*–*89*). Because unvaccinated travelers from nonendemic countries are usually immunologically naïve, travel-associated JE can occur in persons of any age.

### Clinical Manifestations and Diagnosis

#### Signs and Symptoms

The majority of JE virus infections in humans are asymptomatic, and <1% of persons infected with JE virus develop encephalitis (*83*,*90*–*94*). Acute encephalitis is the most commonly identified clinical syndrome among persons with JE virus infection, although milder forms of disease (e.g., aseptic meningitis or undifferentiated febrile illness) also can occur (*6*,*13*,*95*–*97*). Among patients who develop clinical symptoms, the incubation period is 5–15 days. Initial symptoms are usually nonspecific and might include fever, rigors, headache, vomiting, and diarrhea (*6*,*61*,*98*,*99*). Mental status changes, generalized weakness, focal neurologic deficits (e.g., hemiplegia, tetraplegia, or cranial nerve palsies), and movement disorders might occur over the next few days (*61*,*98*–*103*). Seizures are common, especially among children (*61*,*98*–*100*,*103*–*105*). A distinctive clinical presentation of JE is a parkinsonian syndrome resulting from extrapyramidal involvement, with mask-like facies, tremor, cogwheel rigidity, and choreoathetoid movements (*6*,*99*). Acute flaccid paralysis, with clinical and pathological features similar to poliomyelitis, also has been associated with JE virus infection (*6*,*106*,*107*). Status epilepticus, brain hypoxia, increased intracranial pressure, brainstem herniation, and aspiration pneumonia are the most common complications associated with poor outcome and death (*6*,*98*,*104*,*108*).

#### Clinical Laboratory Findings and Neuroimaging

Clinical laboratory findings with JE are nonspecific and might include moderately elevated white blood cell count, mild anemia, and hyponatremia (*6*,*95*,*98*,*99*,*103*). Thrombocytopenia and elevated hepatic enzymes have been reported (*99*). Cerebrospinal fluid (CSF) usually shows a lymphocytic pleocytosis with moderately elevated protein levels (*6*,*59*,*61*,*95*,*98*,*100*,*103*,*109*).

Magnetic resonance imaging (MRI) is the best means for detecting JE-associated abnormalities of the brain, including changes in the thalamus, basal ganglia, midbrain, pons, and medulla (*110*–*112*). Thalamic lesions are the most commonly described abnormality (*110*,*112*).

#### Laboratory Diagnosis

JE virus infections are usually confirmed by detection of virus-specific antibody in CSF or serum (*13*,*113*–*117*). Because humans have low or undetectable levels of viremia by the time the clinical illness occurs, virus isolation and nucleic acid amplification tests (NAATs) are insensitive and should not be used for ruling out a JE diagnosis (*118*,*119*). In one study in Thailand, JE virus could not be isolated from 30 nonfatal JE cases with plasma and CSF samples (*120*). In contrast, JE virus was isolated from CSF from five (33%) of 15 patients who died and from brain tissue from eight (73%) of 11 who died. More recent studies have demonstrated the usefulness of NAAT to diagnose JE in some patients with encephalitis or aseptic meningitis, and JE virus RNA was detected in urine of a patient who died (*97*,*121*,*122*). However, testing by NAAT lacks the sensitivity needed for routine diagnosis.

Acute-phase specimens should be tested for JE virus immunoglobulin M (IgM) antibodies using an IgM antibody-capture enzyme-linked immunosorbent assay (MAC ELISA) (*13*,*113*–*117*). JE virus IgM antibodies can be measured in the CSF of most patients within 4 days of onset of symptoms and in serum by 7–8 days after onset (*76*,*115*,*116*,*123*,*124*). The presence of JE virus IgM antibodies in CSF provides evidence that JE virus infection is the cause of the neurologic illness (*114*,*119*). With clinical and epidemiologic correlation, a positive IgM test has good diagnostic predictive value, although cross-reaction with other flaviviruses can occur.

Plaque reduction neutralization tests (PRNTs) can be performed to confirm recent infection on the basis of a fourfold or higher rise in virus-specific neutralizing antibodies between acute- and convalescent-phase serum specimens or to discriminate between cross-reacting antibodies attributed to another primary flavivirus infection. In patients who have been infected previously by another flavivirus or vaccinated with a flaviviral vaccine (e.g., yellow fever), cross-reactive antibodies in both the ELISA and neutralization assays make identifying a specific etiologic agent difficult.

Vaccination history, date of onset of symptoms, and information regarding other arboviruses known to circulate in the geographic area that might cross-react in serologic assays should be considered when interpreting results. Diagnostic testing for JE is available in some state public health laboratories and at CDC.

#### Treatment and Management

JE treatment consists of supportive care and management of complications. No antiviral agent or specific medication is available to mitigate the effects of JE virus infection (*125*). In controlled clinical trials, clinical outcomes were not improved with corticosteroids, interferon alpha-2a, ribavirin, minocycline, or intravenous immunoglobulin (*126*–*130*). Infection with one JE virus genotype is thought to produce lifelong immunity against all genotypes.

#### Outcome and Sequelae

JE has a case-fatality ratio of 20%–30% (*6*,*52*,*53*,*62*,*68*,*84*,*98*–*100*,*109*,*120*,*127*,*131*,*132*). Some deaths occur after a short fulminant course, whereas others occur after a prolonged coma. Although some motor deficits and movement disorders improve after the acute illness, 30%–50% of JE survivors have neurologic or other sequelae even years later (*6*,*100*,*106*,*127*,*131*–*138*). These include seizures, upper and lower motor neuron weakness, cerebellar and extrapyramidal signs, flexion deformities of the arms, hyperextension of the legs, cognitive deficits, language impairment, psychiatric issues, learning difficulties, and behavioral problems (*6*).

### JE Among Travelers

For most travelers to Asia, the risk for JE is very low but varies on the basis of travel destination, duration, season, activities, and accommodations (*4*,*8*,*11*,*139*). The overall incidence of JE among persons from nonendemic countries who travel to Asia is estimated to be less than one case per 1 million travelers. However, persons who stay for prolonged periods in rural areas with active JE virus transmission might have a risk level similar to that of the susceptible resident population. Travelers on brief trips might be at increased risk if they have extensive outdoor or nighttime exposure in rural areas during periods of active transmission (*140*–*142*). Shorter-term (e.g., <1 month) travelers whose visits are restricted to major urban areas are at minimal risk for JE.

Risk for infection for a traveler cannot be inferred from JE incidence among residents of JE-endemic countries. Very few cases might be reported among the local population because of vaccination or natural immunity from previous infection. However, because JE virus is maintained in an enzootic cycle between animals and mosquitoes, susceptible visitors might still be at risk for infection. JE should be suspected in any patient with evidence of a neurologic infection (e.g., encephalitis, meningitis, or acute flaccid paralysis) who recently has returned from a country in Asia or the western Pacific where JE is endemic.

#### JE Among All Travelers from Nonendemic Countries 

During 1973–2017, a total of 85 JE cases among travelers or expatriates from nonendemic countries were published or reported to CDC (*8**–**10**,**122**,**140**–**174*). About twice as many cases were reported in the most recent 10-year period compared with the three previous 10-year periods: 2008–2017 (n = 34), 1998–2007 (n = 18), 1988–1997 (n = 17), and 1978–1987 (n = 13). This change might relate to increased numbers of travelers and increased testing and reporting of disease cases. Overall, 53 (62%) cases occurred in tourists, 16 (19%) in expatriates, six (7%) in soldiers, and one (1%) in a researcher; the type of travel was unknown in nine (11%) cases. The tourist category included seven persons who were traveling to visit friends and relatives and two students on study-abroad programs. The patients were citizens of 20 different countries. The countries where the infection was most commonly acquired were Thailand (n = 26), Indonesia (n = 13), the Philippines (n = 11), China (n = 9), Vietnam (n = 4), and Japan (n = 4). The countries with the highest number of cases (i.e., Thailand and Indonesia) have high-risk areas but also are destinations with high numbers of tourists. In both countries, tourist beach resort areas can be close to rice fields or rural areas with high mosquito densities (e.g., Phuket, Thailand, and Bali, Indonesia). Among the 76 cases for which age was recorded, the median age was 36 years (range: 5 weeks to 91 years). Overall, 50 (59%) cases occurred among males, and 31 (36%) among females; sex was unknown in four cases (5%). Nineteen (22%) patients recovered fully, 39 (46%) survived but had sequelae, 14 (16%) died, and the outcome was unknown for 13 (15%). None of the patients were known to have received JE vaccine. No cases occurred among business or other shorter-term travelers who visited only urban areas.

#### JE Among U.S. Travelers

Before 1973, at least 300 cases of JE had been reported among U.S. military personnel or their family members (*92*,*93*,*95*,*175*–*179*). During 1973–1992, a total of 11 JE cases were reported among U.S. travelers and military personnel. During 1993–2017, after the first licensure of a JE vaccine in the United States in 1992, a total of 12 cases were reported among U.S. travelers, with a median of zero cases per year (range: 0‒2) (*10*,*143*,*144*,*165*,*173*,*174*). On the basis of 12 reported cases during this 25-year period, and approximately 4–5 million U.S. citizen trips to Asia annually, the overall incidence of JE among U.S. travelers is estimated to be <1 case per million trips to Asia (*180*). Among the 12 cases, three (25%) were in children aged ≤11 years, and the remainder were in adults aged ≥17 years. Eight (67%) cases were in males. Six (50%) patients recovered, three (25%) survived but had sequelae, two (17%) died, and the outcome for one (8%) was unknown. Overall, four (33%) cases occurred in U.S. expatriates living in Asia and eight (67%) in tourists. Duration of travel ranged from 10 days to approximately 3 years, and for eight travelers (67%) was ≥1 month. On the basis of a 2007 study, approximately 20% of U.S. travelers to Asia travel for >30 days; therefore, approximately two thirds of U.S traveler cases occurred among the smaller 20% of higher-risk, longer-term travelers (*181*). Among the four shorter-term travelers, three had traveled for 3 to <4 weeks, and one had traveled for 10 days. One shorter-term traveler spent most of the time in rural areas, two stayed in urban areas but took at least one overnight trip to a rural area, and one had no exposure-related information.

The proportion of U.S. travelers who receive JE vaccine is unknown. However, studies suggest JE vaccination rates are low, even among higher-risk travelers. A 2007 survey of adult travelers on direct flights from the United States to Asia determined that 415 (25%) of 1,691 participants described itineraries for which JE vaccination should have been considered, including 330 (20%) who planned to spend ≥30 days in a JE-endemic country and another 85 (5%) shorter-term travelers who planned to spend at least 50% of their time in JE-endemic rural areas (*181*). Of these higher-risk travelers, 47 (11%) reported receiving at least 1 dose of JE vaccine. Among 164 unvaccinated higher-risk travelers who had visited a health care provider to prepare for their trip, 113 (69%) indicated that their health care provider had not offered or recommended JE vaccine. Results of another survey conducted among a group of U.S. clinical practices that provide pretravel health care indicated that 711 (9%) of 8,289 adults had an increased risk for JE on the basis of planned travel to one or more JE-endemic countries for ≥30 days during the JE virus transmission season with a visit to a rural area included in the itinerary (*182*). Among these 711 persons, 188 (26%) were vaccinated during the pretravel visit, and 11 (2%) had received JE vaccine within the previous 2 years; 512 (72%) were not administered JE vaccine. The main reasons noted for nonadministration included that JE vaccine was not indicated (n = 282; 55%), the patient declined (n = 116; 23%), or insufficient time to complete the vaccination series (n = 85; 17%) (*182*). In these two U.S. studies, 2%–4% of lower-risk travelers had been vaccinated (*181*,*182*).

#### Subclinical JE Virus Infection Among Travelers

Two studies have investigated the frequency of subclinical JE virus infection. Among 1,000 unvaccinated U.S. infantry soldiers deployed to Korea for at least 330 days during 2008–2011, predeployment and postdeployment serologic testing suggested one possible subclinical infection (*183*). In a study of 387 Australian adult travelers not vaccinated at their pretravel visit who traveled to Asia for a median of 21 days (range: 7–326 days) and had pretravel and posttravel serologic testing, no JE virus infections occurred; therefore, the risk for subclinical infection was zero per 10,000 traveler-days (95% confidence interval [CI] = 0–3.9) (*184*).

### JE Vaccines

Four types of JE vaccines are manufactured and available in different countries, including a live attenuated vaccine, a live recombinant (chimeric) vaccine, inactivated mouse brain–derived vaccines, and inactivated Vero cell culture–derived vaccines (*3*,*7*,*185*,*186*). JE-VC, manufactured as Ixiaro, is the only JE vaccine licensed and available in the United States. JE-MB, manufactured as JE-VAX, was previously available in the United States; production has ceased, and all doses expired in May 2011 (*16*).

### Correlates of Protection

Because several effective JE vaccines are available in Asia, randomized, controlled efficacy trials to evaluate new JE vaccines would be logistically difficult and potentially unethical. JE-VC was licensed based on its ability to induce JE virus neutralizing antibodies, which is thought to be a reliable surrogate of efficacy (*187*,*188*). Observations from the 1930s indicated that laboratory workers who had been accidentally exposed to JE virus were protected from disease when they had measurable neutralizing antibodies (*187*). These observations were further supported by passive antibody transfer and active vaccination studies in animals using both licensed and experimental JE vaccines. Studies in mice indicated that passive transfer of neutralizing antibodies protected animals against JE virus challenge and established a dose-response relationship between antibody titer and level of protection (*189*–*192*). These studies also indicated that animals that were actively primed but had no detectable neutralizing antibodies against JE virus were protected from lethal challenge, demonstrating an effective anamnestic immune response (*192*). A more recent study indicated that hyperimmune ascitic fluid raised against two JE vaccines derived from genotype III JE virus strains (i.e., JE-MB derived from the Nakayama strain and a chimeric vaccine derived from the SA14-14-2 strain) protected mice against intracerebral challenge with JE virus strains of four genotypes. These data demonstrate that neutralizing antibodies provide protection against heterologous JE virus genotypes (*193*). In another study, mice were passively vaccinated with pooled sera with varying titers of neutralizing antibodies against JE virus from humans vaccinated with JE-VC. Mice were challenged 18 hours later with a lethal dose of either a genotype I (KE-093) or genotype III (SA14) JE virus strain (*194*). Mice with ex vivo neutralizing antibody titers of ≥10 had survival rates of 100% (10 of 10) and 90% (nine of 10) after challenge with the genotype I and III JE virus strains, respectively. In mice receiving lower titer sera, survival correlated with the neutralizing antibody titer of the immunizing sera. Mice actively vaccinated with varying doses of JE-VC and JE-MB also had dose-dependent protection against intraperitoneal challenge with the JE virus SA14 strain (*194*). Finally, in a study designed to develop a JE animal model in nonhuman primates, 16 rhesus macaques were given an intranasal challenge with a 90% effective dose of JE virus (i.e., a dose that when administered via intranasal challenge would be expected to cause encephalitis in 90% of the animals), including four monkeys that were given four doses of an inactivated mouse brain–derived JE vaccine, eight monkeys immunized with one of two developmental poxvirus JE vaccines, and four JE virus–naïve control monkeys (*195*,*196*). The minimum neutralizing antibody titer required to protect the monkeys from lethal challenge was between 30 and 46. The higher titers required for protection in this study might have been caused by the high challenge dose used to develop the model.

PRNT is used to measure functional antibody that inactivates or neutralizes virus. A PRNT_50_ titer is the reciprocal of the endpoint serum dilution that reduces the challenge virus plaque count by 50%. A WHO expert panel accepted a PRNT_50_ titer of ≥10 as an immunologic correlate of protection against JE in humans (*188*). Although a correlate of protection has been defined, a vaccinated person whose neutralizing antibody titer has waned to a level of <10 might still be protected because of persisting immunologic memory. Several studies have shown that vaccinated persons without measurable neutralizing antibodies can mount a rapid anamnestic response to infection (*197*–*199*). T cells likely also play a key role in clearing JE virus.

PRNT can be performed using various protocols, and the validity and comparability of PRNT results depend on detailed components of the selected assay (e.g., endpoint neutralization, incubation conditions, cell substrate, and target virus) (*188*,*200*). Although JE virus PRNTs are performed only at selected reference laboratories, careful attention must be paid to the characteristics and validation of a PRNT assay that is used to measure JE virus neutralizing antibody titers as a surrogate for efficacy.

## JE-VC

### Manufacture and Licensure

In March 2009, FDA approved JE-VC for use in persons aged ≥17 years; in May 2013, licensure was extended to include children aged ≥2 months (*17*). The booster dose was approved for persons aged ≥17 years in October 2010 and for children in April 2018.

JE-VC is an inactivated vaccine derived from the attenuated SA14-14-2 JE virus strain propagated in Vero cells (Box 1) (*14*,*201*,*202*). Each 0.5-mL dose contains 6 antigen units of purified, inactivated JE virus and approximately 250 *μ*g aluminum hydroxide as an adjuvant.

BOX 1 **Composition, storage, dose, and administration of inactivated Vero cell culture–derived Japanese encephalitis vaccine**

**Trade name:** Ixiaro

**JE virus strain**: SA14-14-2

**JE virus seed**: Attenuated

**Substrate**: Vero cells

**Adjuvant**: Aluminum hydroxide

**Stabilizer**: None

**Preservative**: None

**Final preparation**:* Liquid

**Storage**: 35°F–46°F (2°C–8°C)

**Presentation**: Prefilled syringe

**Route**: Intramuscular

**Dose and schedule, by age group:**

- 2–35 months: 2 doses (0.25 mL each) administered on days 0 and 28 ^†^
- 3–17 years: 2 doses (0.5 mL each) administered on days 0 and 28
- 18–65 years: 2 doses (0.5 mL each) administered on days 0 and 7–28^§^
- >65 years: 2 doses (0.5 mL each) administered on days 0 and 28

**Booster dose, by age group (if ongoing exposure or reexposure is expected):**

- <3 years: 1 dose (0.25 mL) at ≥1 year after the second dose ^†,¶^
- ≥3 years: 1 dose (0.5 mL) at ≥1 year after the second dose^¶^

*The final preparation contains residues of protamine sulfate.

^†^ To administer a 0.25-mL dose, expel and discard half of the volume from the 0.5-mL prefilled syringe.

^§^ Studies showing the second dose can be administered as early as 7 days after the first dose only included adults aged 18–65 years.

^¶^ No data are available on the response to a booster dose administered >2 years after the second dose.

### Immunogenicity of JE-VC in Adults

#### Primary Series at 0 and 28 Days

No efficacy data exist for JE-VC. The vaccine was licensed on the basis of its ability to induce JE virus neutralizing antibodies as a surrogate for protection. The pivotal noninferiority immunogenicity study compared 2 doses of JE-VC given on days 0 and 28 to 3 doses of JE-MB given on days 0, 7, and 28 to adults aged ≥18 years in the United States, Austria, and Germany (*203*). Prevaccination PRNT_50_ titers were <10 for all participants. In the per-protocol analysis, 352 (96%) of 365 JE-VC recipients developed a PRNT_50_ titer ≥10, compared with 347 (94%) of 370 JE-MB recipients at 28 days after the last dose (*14*). PRNT_50_ titers were >80 among 91% of the 361 JE-VC recipients for whom data were available. The difference in seroconversion rates was 2.6% (95% CI = −0.5%–6%), and noninferiority of JE-VC compared with JE-MB was established (*14*,*203*). The PRNT_50_ geometric mean titer (GMT) for JE-VC recipients was 244 (95% CI = 216–274), compared with 102 (95% CI = 90–115) for JE-MB recipients. However, the target JE virus strain in the neutralizing antibody assay was SA14-14-2 (i.e., the JE virus strain used in JE-VC), whereas JE-MB is produced from the Nakayama JE virus strain. The GMT ratio was 2.3 (95% CI = 2.0–2.8), and noninferiority was again established.

The licensed vaccine schedule was derived in part from a study that compared 2 6-*μ*g doses of vaccine administered 28 days apart to a single dose of either 6 *μ*g or 12 *μ*g (*204*). Twenty-eight days after receiving 1 dose of the standard 6-*μ*g regimen, only 95 (41%) of 230 JE-VC recipients had seroconverted with a PRNT_50_ titer ≥10. Fifty-six days after receiving their first dose of vaccine, 110 (97%) of 113 participants who had received 2 doses had a PRNT_50_ titer ≥10, compared with 30 (26%) of 117 and 47 (41%) of 114 of those who received a single 6-*μ*g or 12-*μ*g dose, respectively; GMTs in the three groups were 218, 8, and 11, respectively. All of the 2-dose recipients who seroconverted had protective antibodies by 7 days after receiving the second dose of vaccine. In all other prelicensure and postlicensure randomized controlled trials and observational studies, ≥95% of adults were seroprotected after receiving 2 doses of JE-VC administered 28 days apart, with the exception of one study that showed lower seroprotection rates among adults aged ≥64 years (Table 1) (*201*,*203*–*209*).

**TABLE 1:** Seroprotection rates at 1 month after a 2-dose primary series of inactivated Vero cell culture–derived Japanese encephalitis vaccine administered according to the dose and schedule approved by the Food and Drug Administration, by age group

Age group (yrs)	Study location	Seroprotection rate*			Reference
		Total	No. seroprotected	(%)	
≥18	United States, Europe	**361**	352	98	203
≥18	Europe	**127**	126	99	209
≥18	Europe	**113**	110	97	204
≥18	Europe	**31**	30	97^†^	208
≥18	United States	**92**	88	96	207
18–49	United States	**22**	21	95	201
18–65	Europe	**206**	206	100	205
≥64	Europe	**197**	128	65	206

* Proportion with 50% plaque reduction neutralization test titer ≥10.

^†^ Seroprotection measured 4–8 weeks after dose 2.

#### Adults Aged ≥65 Years

Prelicensure clinical trials did not include sufficient numbers of persons aged ≥65 years to allow an adequate assessment of immunogenicity in this age group. One immunogenicity study of JE-VC included 24 persons aged ≥65 years who received the 2-dose primary series per protocol. At 28 days after the second dose, 23 (96%) persons had a seroprotective titer, and the GMT was 255 (*14*,*203*).

One postlicensure phase IV observational study was conducted to investigate immunogenicity of JE-VC in older adults (*206*). The median age was 69 years (range: 64–83 years). Forty-two days after the second dose of a 2-dose primary series, 128 (65%) of 197 persons were seroprotected and the GMT was 37. Both the seroprotection rate and GMT were substantially lower compared with results in the pivotal immunogenicity study of JE-VC in which study participants had a median age of 41 years (*203*). Seroprotection after the second dose was measured at 42 days in the study among older adults compared with 28 days in the pivotal immunogenicity study; however, that difference is unlikely to explain the results. In a subanalysis of 173 persons aged 65–74 years compared with 23 persons aged 75–83 years, the seroprotection rates and GMTs were similar in both of these groups. No data were gathered on seroprotection rates at >42 days after the second dose, or immunologic response to an additional dose or early booster dose of JE-VC.

#### Delayed Administration of the Second Dose of the Primary Series

In one study, persons who had previously received a single 6-*μ*g dose of JE-VC and had a PRNT_50_ titer <10 at month 6 received a second 6-*μ*g dose at month 11 (*14*,*210*). At 28 days after the second dose, 99 (99%) of 100 persons were seroprotected, and the GMT was 504 (95% CI = 367‒692). Compared with 2 doses administered at a 28-day interval, 2 doses administered at an interval of 11 months resulted in a similar rate of seroprotection and a higher GMT. At 13 months after dose 2 of the 0- and 11-month schedule, 85 (89%) of 96 participants were still seroprotected, and the GMT was 121 (95% CI = 87‒168). Other than anecdotal reports of a small number of study participants who seroconverted when 2 doses were administered 23 months apart, no data are available on the immunogenicity of the primary series administered at an interval of >11 months.

#### Accelerated Primary Series in Adults Aged 18–65 years

A randomized, controlled trial in adults aged 18–65 years in Austria, Germany, and Switzerland investigated immunogenicity after JE-VC administered in an accelerated primary schedule on days 0 and 7, given concomitantly with purified chick embryo cell rabies vaccine (*14*,*205*). In two comparison groups, JE-VC was administered according to a conventional 2-dose primary series on days 0 and 28, with or without rabies vaccine. Rabies vaccine was administered in the accelerated schedule group on days 0, 3, and 7 (an unlicensed regimen in the United States) and in the conventional group on days 0, 7, and 28. Twenty-eight days after the second JE-VC dose, 203 (99%) of 206 persons in the accelerated schedule group, 157 (100%) of 157 persons in the JE-VC conventional schedule with rabies vaccine group, and 49 (100%) of 49 persons in the JE-VC conventional schedule alone group were seroprotected (Table 2). The PRNT_50_ GMT in the accelerated schedule group was 690 compared with 299 for the conventional JE-VC schedule with rabies vaccine and 337 for the conventional JE-VC schedule alone. At 10–12 months after the second dose, seroprotection rates were 94% for the accelerated schedule group and 86% and 88% for the other two groups (*14*,*211*). The GMT in the accelerated schedule group was 117, threefold higher than the GMTs of 39 in the other two groups. The reason for the higher GMT in the accelerated schedule group is unknown; no study participants reported vaccination with other flavivirus vaccines during the study period (*211*).

**TABLE 2:** Seroprotection rates and geometric mean titers for inactivated Vero cell culture–derived Japanese encephalitis vaccine administered to adults aged 18–65 years in an accelerated schedule with rabies vaccine or standard schedule with and without rabies vaccine*

Measure and time after second JE-VC dose	Primary series schedule								
	JE-VC, 0 and 7 days with rabies vaccine^†^			JE-VC, 0 and 28 days with rabies vaccine^§^			JE-VC, 0 and 28 days alone		
**Seroprotection rate^¶^**	**Total**	**No. seroprotected**	**(%)**	**Total**	**No. seroprotected**	**(%)**	**Total**	**No. seroprotected**	**(%)**
28 days	**206**	203	99	**157**	157	100	**49**	49	100
>300 days**	**199**	188	94	**154**	132	86	**48**	42	88
**GMT^††^**	**GMT (95% CI)**			**GMT (95% CI)**			**GMT (95% CI)**		
28 days	690 (595‒801)			299 (254‒352)			337 (252‒451)		
>300 days**	117 (100‒137)			39 (33‒47)			39 (28‒54)		

**Sources:** Food and Drug Administration. Ixiaro: Japanese encephalitis vaccine, inactivated, adsorbed [package insert]. Vienna, Austria: Valneva Austria GmbH; 2018. https://www.fda.gov/media/75777/download; Jelinek T, Burchard GD, Dieckmann S, et al. Short-term immunogenicity and safety of an accelerated pre-exposure prophylaxis regimen with Japanese encephalitis vaccine in combination with a rabies vaccine: A phase III, multicenter, observer-blind study. J Travel Med 2015;22:225–31; Cramer JP, Jelinek T, Paulke-Korinek M, et al. One-year immunogenicity kinetics and safety of a purified chick embryo cell rabies vaccine and an inactivated Vero cell-derived Japanese encephalitis vaccine administered concomitantly according to a new, 1-week, accelerated primary series. J Travel Med 2016;23:1–8.

**Abbreviations:** CI = confidence interval; GMT = geometric mean titer; JE-VC = Vero cell culture–derived Japanese encephalitis vaccine; PCEC = purified chick embryo cell.

* Per-protocol analysis.

^†^ PCEC rabies vaccine administered in a 0-, 3-, 7-day schedule.

^§^ PCEC rabies vaccine administered in a 0-, 7-, 28-day schedule.

^¶^ Proportion with 50% plaque reduction neutralization test titer ≥10.

** Study ended on day 365.

^††^ PRNT titers <10 were imputed to 5.

Data on a shorter schedule also are available from a phase II study of JE-VC that investigated alternate dosing schedules among adults aged 18–49 years (*201*). One study arm included persons who received JE-VC on a 0-, 14- and 28 -day schedule, and a blood sample was collected before vaccination on day 28. At 14 days after administration of the 0- and 14-day doses, 22 (96%) of 23 persons were seroprotected, and the GMT was 328 (95% CI = 189‒570).

#### Adults with Preexisting Flavivirus Antibodies

A study that evaluated the effect of preexisting antibodies against tickborne encephalitis (TBE) virus, another flavivirus, determined that TBE virus antibodies enhanced the response to JE-VC after the first dose but had no effect after the 2-dose primary series (*212*). After 1 dose of JE-VC, 62 (77%) of 81 persons with preexisting TBE virus IgG antibodies developed protective antibodies against JE virus compared with 166 (49%) of 339 JE-VC recipients with no preexisting TBE virus antibodies. However, after the second dose of JE-VC, persons with and without TBE virus antibodies had similarly high rates of seroprotection against JE virus, with 78 (96%) of 81 and 310 (91%) of 339, respectively, with protective antibodies; this difference was not statistically significant (p = 0.17). JE virus PRNT_50_ GMTs also were similar between the groups after 2 doses of JE-VC (207 and 187, respectively; p = 0.56).

#### Duration of Neutralizing Antibodies After JE-VC Primary Series

Three clinical trials provided data on persistence of protective neutralizing antibodies after a primary JE-VC series of 2 doses administered 28 days apart. In a study performed in central Europe (Austria, Germany, and Romania), seroprotection rates ranged from 95% at 6 months to 82% at 60 months after receiving the first dose of the 2-dose series (Table 3) (*14*,*213*,*214*). A study that used similar methods but was performed in western and northern Europe (Germany and Northern Ireland) found that among adults receiving 2 doses of JE-VC, seroprotection rates were 83% (96 of 116) at 6 months, 58% (67 of 116) at 12 months, and 48% (56 of 116) at 24 months after their first vaccination (*165*). In a third clinical trial, conducted in Austria and Germany, at 15 months after the first dose of the 2-dose JE-VC vaccination series, 69% (137 of 198) of participants had a protective neutralizing antibody titer (*215*).

**TABLE 3:** Seroprotection rates and geometric mean titers among adults at intervals after the first dose of a 2-dose primary series of inactivated Vero cell culture–derived Japanese encephalitis vaccine

Measure and study site	6 mos			12**‒**15 mos			24 mos			60 mos		
Seroprotection rate*	**Total**	**No. seroprotected**	**(%)**	**Total**	**No. seroprotected**	**(%)**	**Total**	**No. seroprotected**	**(%)**	**Total**	**No. seroprotected**	**(%)**
Austria, Germany, Romania	181	172	(95)	181	151	(83)	181	148	(82)	151	124	(82)
Germany, Northern Ireland	116	96	(83)	116	67	(58)	116	56	(48)	—	—	—
Austria, Germany	—	—	—	198	137	(69)	—	—	—	—	—	—
**GMT**	**GMT (95% CI)**			**GMT (95% CI)**			**GMT (95% CI)**			**GMT (95% CI)**		
Austria, Germany, Romania	84 (71‒98)			41 (34‒49)			44 (37‒53)			43 (36‒53)		
Germany, Northern Ireland	47 (37‒59)			18 (14‒23)			16 (13‒21)			—		
Austria, Germany	—			23 (19‒27)			—			—		

**Sources:** Food and Drug Administration. Ixiaro: Japanese encephalitis vaccine, inactivated, adsorbed [package insert]. Vienna, Austria: Valneva Austria GmbH; 2018. https://www.fda.gov/media/75777/download; Dubischar-Kastner K, Eder S, Buerger V, et al. Long-term immunity and immune response to a booster dose following vaccination with the inactivated Japanese encephalitis vaccine IXIARO, IC51. Vaccine 2010;28:5197–202. Schuller E, Jilma B, Voicu V, et al. Long-term immunogenicity of the new Vero cell-derived, inactivated Japanese encephalitis virus vaccine IC51 Six and 12 month results of a multicenter follow-up phase 3 study. Vaccine 2008;26:4382–6. Dubischar-Kastner K. New clinical data for IXIARO Japanese encephalitis vaccine, inactivated, adsorbed. Presentation to Advisory Committee on Immunization Practices (ACIP), February 24, 2016. Atlanta, GA: US Department of Health and Human Services, CDC; 2016. https://stacks.cdc.gov/view/cdc/60592; Eder S, Dubischar-Kastner K, Firbas C, et al. Long term immunity following a booster dose of the inactivated Japanese Encephalitis vaccine IXIARO®, IC51. Vaccine 2011;29:2607–12.

**Abbreviations:** CI = confidence interval; GMT = geometric mean titer.

* Proportion with 50% plaque reduction neutralization test titer ≥10.

To investigate possible reasons for the substantially different seroprotection rates at similar time points in the three studies, a subsequent analysis was conducted using participant data from the study conducted in central Europe (Austria, Germany, and Romania) and stratifying participants by TBE vaccination status (Table 4) (*214*,*216*). In the stratified analysis, seroprotection rates were lower at all time points from 6 to 60 months after the first dose of a 2-dose primary series in the group that had not received TBE vaccine compared with the group with persons who had received TBE vaccine before or during the study. In the United States, TBE vaccine is not available, and other flavivirus vaccines are not routinely administered with JE-VC; therefore, the immunologic response after JE-VC is likely to be most similar to the participants who did not receive the TBE vaccine.

**TABLE 4:** Seroprotection rates and geometric mean titers among adults at intervals after first dose of a 2-dose primary series of inactivated Vero cell culture–derived Japanese encephalitis vaccine, by tickborne encephalitis vaccination status

Measure	6 mos			12 mos			24 mos			60 mos		
Seroprotection rate*	**Total**	**No. seroprotected**	**(%)**	**Total**	**No. seroprotected**	**(%)**	**Total**	**No. seroprotected**	**(%)**	**Total**	**No. seroprotected**	**(%)**
TBE vaccine^†^	**89**	86	(97)	**89**	82	(92)^§^	**86**	78	(91)^§^	**78**	67	(86)^§^
No TBE vaccine	**92**	86	(93)	**92**	69	(75)	**78**	53	(68)	**47**	30	(64)
**GMT**	**GMT**			**GMT**			**GMT**			**GMT**		
TBE vaccine^†^	96			48			56			45		
No TBE vaccine	73			35			33			29		

**Sources:** Dubischar-Kastner K. New clinical data for IXIARO Japanese encephalitis vaccine, inactivated, adsorbed. Presentation to Advisory Committee on Immunization Practices (ACIP), February 24, 2016. Atlanta, GA: US Department of Health and Human Services, CDC; 2016. https://stacks.cdc.gov/view/cdc/60592; Taucher C, Kollaritsch H, Dubischar KL. Persistence of the immune response after vaccination with the Japanese encephalitis vaccine, IXIARO® in healthy adults: A five year follow-up study. Vaccine 2019;37:2529–31.

**Abbreviations:** GMT = geometric mean titer; TBE = tickborne encephalitis.

* Proportion with 50% plaque reduction neutralization test titer ≥10.

^†^ TBE vaccine received before or after Vero cell culture–derived Japanese encephalitis vaccine.

^§^ Nonoverlapping 95% confidence intervals (calculated according to the method recommended by Altman, developed by Wilson) for seroprotection rates for TBE and no TBE vaccine groups.

#### Immunologic Response After a Booster Dose

Two clinical trials provided data on the response to a booster dose of JE-VC. In a study conducted in Austria and Germany, 198 adults aged ≥18 years who had received a 2-dose primary series of JE-VC were administered a booster dose 15 months after the first dose (*215*). The percentage of participants with a protective neutralizing antibody titer increased from 69% (137 of 198) before the booster dose to 100% (198 of 198) at 28 days after the booster dose, and a protective titer was found in 98% of persons at 6 months and 12 months after the booster dose (Table 5). The GMT before the booster was 23 and increased fortyfold to 900 at 28 days after the booster dose. At approximately 76 months after the booster dose, 64 (96%) of 67 participants still had a PRNT_50_ titer ≥10 and the GMT was 148, indicating good seroprotection rates for at least 6 years after a booster dose (*217*). GMTs at month 76 were not significantly different in persons with and without a history of TBE or yellow fever vaccination. Data from this study were used in a mathematical model to estimate that protection after the booster dose would last an average of 14 years (range: 2–25 years).

**TABLE 5:** Seroprotection rates and geometric mean titers before and after a booster dose of inactivated Vero cell culture–derived Japanese encephalitis vaccine administered 15 months after the first dose of a 2-dose primary series

Measure	0 days			1 mo			6 mos			12 mos			76 mos		
Seroprotection rate*	**Total**	**No. seroprotected**	**(%)**	**Total**	**No. seroprotected**	**(%)**	**Total**	**No. seroprotected**	**(%)**	**Total**	**No. seroprotected**	**(%)**	**Total**	**No. seroprotected**	**(%)**
	198	**137**	**(69)**	198	**198**	**(100)**	197	**194**	**(98)**	194	**191**	**(98)**	67	**64**	**(96)**
**GMT**	**GMT (95% CI)**			**GMT (95% CI)**			**GMT (95% CI)**			**GMT (95% CI)**			**GMT (95% CI)**		
	23 (19–27)			900 (742–1,091)			487 (391–608)			361 (295–444)			148 (107–207)		

**Sources:** Eder S, Dubischar-Kastner K, Firbas C, et al. Long term immunity following a booster dose of the inactivated Japanese Encephalitis vaccine IXIARO®, IC51. Vaccine 2011;29:2607–12; Paulke-Korinek M, Kollaritsch H, Kundi M, Zwazl I, Seidl-Friedrich C, Jelinek T. Persistence of antibodies six years after booster vaccination with inactivated vaccine against Japanese encephalitis. Vaccine 2015;33:3600–4.

**Abbreviations:** CI = confidence interval; GMT = geometric mean titer.

* Proportion with 50% plaque reduction neutralization test titer ≥10.

In a second study, a booster dose was administered to 40 persons who had received a 2-dose primary series but no longer had protective neutralizing antibody titers (*210*). The booster was administered at 11 months (n = 16) or 23 months (n = 24) after the first dose and resulted in protective titers in all persons. GMTs at 1 month after the booster increased to 676 (95% CI = 365‒1,253) in the group administered the dose at 11 months and 2,496 (95% CI = 1,408‒4,427) in those vaccinated at 23 months after the primary series. Among the 16 persons who received the booster dose at 11 months, all still had seroprotective titers 13 months later.

#### Use of JE-VC After Primary Vaccination with JE-MB

In a study among U.S. military personnel who had received at least 3 doses of JE-MB or were JE vaccine naïve, persons were vaccinated with 2 doses of JE-VC on days 0 and 28 and immunogenicity was assessed at 28 days after 1 dose in the previously JE-MB-vaccinated persons and 2 doses in the vaccine-naïve persons (*207*). The previously JE-vaccinated persons had received their last JE-MB dose a median of 2.9 years earlier (range: 1.8–10.2 years). In the per-protocol analysis, the seroprotection rate among previously vaccinated participants on day 28 after 1 dose of JE-VC was 100% (44 of 44) and in previously unvaccinated participants at 28 days after 2 doses was 93% (53 of 57). The GMT was significantly higher in previously vaccinated participants after 1 dose (GMT 315; 95% CI = 191–520) compared with the previously unvaccinated participants after 2 doses (GMT 79; 95% CI = 54–114). Among previously JE-vaccinated persons, the time since receiving their last dose did not significantly affect the neutralizing antibody titers achieved after 1 dose of JE-VC; however, only 12 (27%) participants had received their last dose of JE-MB ≥5 years before enrollment.

In another U.S. study using archived sera from military personnel, immunogenicity at 12–23 months after a single dose of JE-VC was assessed in adults previously vaccinated with at least 3 doses of JE-MB compared with JE vaccine-naïve adults vaccinated with a 2-dose JE-VC series (*218*). Persons with a history of JE-MB vaccination had received their last JE-MB dose a median of 2.9 years (range: 1 day–19 years) before the JE-VC dose and had received a median of three JE-MB doses. At 12–23 months, seroprotection rates were 94% (235 of 250) in previously JE-MB-vaccinated personnel and 54% (135 of 250) in previously unvaccinated personnel. The GMT of 75 (95% CI = 63–90) in the previously JE-MB-vaccinated personnel was significantly higher than the GMT of 12 (95% CI = 11–14) in the previously unvaccinated personnel.

An observational study was conducted at travel clinics in Scandinavia among adults planning travel to a JE-endemic area (*208*). One study cohort included participants who had received 2 or 3 doses of JE-MB and were vaccinated with 1 dose of JE-VC; the comparison group included JE vaccine-naïve persons who were vaccinated with 2 doses of JE-VC on days 0 and 28. Among the previously vaccinated persons who received 1 dose of JE-VC, their last JE-MB dose was a median of 5.2 years earlier (range: 1‒21 years). At 4‒8 weeks after the JE-VC dose, 98% (41 of 42) were seroprotected with a GMT of 504. Among unvaccinated persons, 4‒8 weeks after two JE-VC doses, 97% (30 of 31) were seroprotected with a GMT of 499. In a follow-up study investigating duration of protection, only 47% of persons from the original study cohorts were available (*219*). A mean of 2.1 years after the final JE-VC dose, the seroprotection rate among previously JE-MB-vaccinated persons who had received 1 dose of JE-VC was 100% (18 of 18) and among previously unvaccinated persons who had received 2 doses of JE-VC was 93% (14 of 15).

The results of these studies indicate that among persons who previously received JE-MB vaccine, a single dose of JE-VC results in seroprotection rates and GMTs at approximately 4 weeks that are noninferior to those in unvaccinated persons who receive a standard 2-dose JE-VC series. At 12–23 months, the immunological response after 1 JE-VC dose in adults previously vaccinated with at least 3 doses of JE-MB remains noninferior to the response in JE vaccine-naïve adults vaccinated with the 2-dose primary series of JE-VC.

#### Concomitant Administration of JE-VC with Other Vaccines

##### JE-VC with Hepatitis A Vaccine

A clinical trial in which the first dose of JE-VC was administered concomitantly with hepatitis A vaccine indicated no interference with the immune response to JE-VC or hepatitis A vaccine (*209*). Among the 58 persons who received both JE-VC and hepatitis A vaccine in the per-protocol analysis, all had protective neutralizing antibodies compared with 98% (57 of 58) of persons who received JE-VC alone. GMTs also were similar at 203 (95% CI = 154–261) and 192 (95% CI = 148–250), respectively. In addition, persons receiving JE-VC and hepatitis A vaccine had similar seroconversion rates for antihepatitis A virus (anti-HAV) antibody (100%, 58 of 58) compared with persons receiving hepatitis A vaccine alone (96%, 50 of 52) and HAV antibody GMTs were similar (150 and 124, respectively). However, some differences were noted between men and women in the levels of anti-HAV antibody achieved, and both seroconversion rates and antibody titers varied depending on which anti-HAV assay was used; whether these observations have any clinical significance is not known.

##### JE-VC with Rabies Vaccine

A randomized trial evaluated immunologic responses to JE-VC and a purified chick embryo cell culture rabies vaccine when vaccines were administered alone or concomitantly to adults aged 18‒65 years (*14*,*205*,*220*). JE-VC was administered in a 2-dose schedule on days 0 and 28 and rabies vaccine in a 3-dose schedule on days 0, 7, and 28. Twenty-eight days after the second JE-VC dose, all persons in the concomitant administration group (157/157) and in the group administered JE-VC alone (49 of 49) had seroprotective neutralizing antibody titers against JE virus and GMTs were similar at 299 and 337, respectively (Table 2). At 12 months after the first JE-VC dose, JE seroprotection rates were similar at 86% (132 of 154) and 88% (42 of 48), and the GMT for both groups was 39 (*211*). The percentage of persons with protective neutralizing antibody concentrations against rabies virus (i.e., ≥0.5 IU/mL) at 28 days after the third rabies vaccine dose was 100% (157 of 157) in the concomitant administration group and 99% (203 of 204) in the group administered rabies vaccine alone (*220*). Noninferiority of the immunologic responses to JE-VC and rabies vaccine was established for concomitant administration compared with separate administration of either vaccine.

##### JE-VC and Rabies Vaccine with Meningococcal Vaccine

Another study was conducted in which JE-VC and purified chick embryo cell rabies vaccine were administered concomitantly, with or without a quadrivalent meningococcal conjugate vaccine, to adults aged 18–60 years (*221*). Two additional groups received rabies vaccine alone or meningococcal vaccine alone. JE-VC was administered according to a 0- and 28-day schedule, rabies vaccine on a 0-, 7-, and 28-day schedule, and meningococcal vaccine as a single dose on day 0. In the per-protocol analysis, at 28 days after the final doses of JE-VC and rabies vaccine, 95 (98%) of 97 adults who received all three vaccines had seroprotective titers against JE virus, compared with 95 (99%) of 96 persons who received JE-VC and rabies vaccine. GMTs also were similar at 165 (95% CI = 136‒199) and 183 (95% CI = 151‒221), respectively. All persons in the three groups that received rabies vaccine developed protective rabies virus antibody concentrations of ≥0.5 IU/mL. Measurement of the response to meningococcal vaccine was at 56 days in persons who received the three vaccines concomitantly and at 28 days in the group that received meningococcal vaccine alone. No significant differences were found in the percentage of participants who achieved human serum bactericidal assay antibody titers ≥1:8 against meningococcal serogroups A, C, W, or Y; however, the GMT against each serogroup was higher when meningococcal vaccine was administered alone. Interpretation of these results is complicated by the different time points of the blood draws for the two groups.

#### Immunogenicity of JE-VC Against Different JE Virus Genotypes

JE-VC is derived from the genotype III SA14-14-2 JE virus. To assess cross-protection provided against different JE virus genotypes, a study was conducted among European travelers who were administered a 2-dose primary JE-VC series and evaluated for the presence of neutralizing antibodies against JE virus strains representing genotypes I, II, III, and IV (*222*). At 4–8 weeks after the primary series, among 29 persons vaccinated with JE-VC, ≥93% had PRNT titers ≥10 against each of the virus strains. GMTs ranged from 55 for the genotype I strain to 811 for the genotype II strain.

### Immunogenicity of JE-VC in Children

#### Primary Series

The pivotal pediatric clinical trial of JE-VC was conducted among children aged 2 months–17 years in the Philippines (*14*,*223*). Among children randomly assigned to receive 2 age-appropriate doses of JE-VC, 384 (99.7%) of 385 were seroprotected at 28 days after the second dose (95% CI = 96%–100%) (Table 6). Seroprotection rates were similar in children who received the 0.25-mL and 0.5-mL doses.

**TABLE 6:** Seroprotection rates in children at 1 month after a 2-dose primary series of inactivated Vero cell culture–derived Japanese encephalitis vaccine administered according to the dose and schedule approved by the Food and Drug Administration*

Study site	Age group	Seroprotection rate^†^					
		0.25-mL JE-VC dose			0.5-mL JE-VC dose		
		Total	No. seroprotected	(%)	Total	No. seroprotected	(%)
Philippines	2 mos–17 yrs	**148**	147	(99)^§^	**237**	237	(100)
India	1–2 yrs	**23**	22	(96)	**—**	—	—^¶^
United States, Europe, Australia	2 mos–17 yrs	**5**	5	(100)	**57**	57	(100)

**Sources:** Food and Drug Administration. Ixiaro: Japanese encephalitis vaccine, inactivated, adsorbed [package insert]. Vienna, Austria: Valneva Austria GmbH; 2018. https://www.fda.gov/media/75777/download; Dubischar KL, Kadlecek V, Sablan JB, et al. Immunogenicity of the inactivated Japanese encephalitis virus vaccine IXIARO in children from a Japanese encephalitis virus-endemic region. Pediatr Infect Dis J 2017;36:898–904; Kaltenböck A, Dubischar-Kastner K, Schuller E, Datla M, Klade CS, Kishore TS. Immunogenicity and safety of IXIARO (IC51) in a Phase II study in healthy Indian children between 1 and 3 years of age. Vaccine 2010;28:834–9; Jelinek T, Cromer MA, Cramer JP, et al. Safety and immunogenicity of an inactivated Vero cell–derived Japanese encephalitis vaccine (IXIARO®, JESPECT®) in a pediatric population in JE non-endemic countries: An uncontrolled, open-label phase 3 study. Travel Med Infect Dis 2018;22:18–24.

**Abbreviations:** FDA = Food and Drug Administration; JE-VC = Vero cell culture–derived Japanese encephalitis vaccine.

* For children aged 2 months–2 years, 2 doses (0.25 mL each) administered 28 days apart; for children aged 3–17 years, 2 doses (0.5 mL each) administered 28 days apart.

^†^ Proportion with 50% plaque reduction neutralization test titer ≥10.

^§^ Of an additional 98 children aged 3–11 years who received 2 doses of 0.25 mL, 94 (96%) were seroprotected at 1 month after the second dose.

^¶^ Of 21 children aged 1–2 years who received 2 doses of 0.5 mL, 20 (95%) were seroprotected at 1 month after the second dose; the FDA-approved dose for children aged 1–2 years is 0.25 mL.

In a randomized, controlled trial conducted in India among children aged 1–2 years, 22 (96%) of 23 children (95% CI = 87%–100%) were seroprotected at 28 days after receiving 2 0.25-mL doses of JE-VC compared with 10 (91%) of 11 (95% CI = 74%–100%) children who received 3 doses of an inactivated mouse brain–derived JE vaccine (*14*,*224*). GMTs were 201 (95% CI = 106–380) and 230 (95% CI = 68–784), respectively. In an observational study of 62 children from countries without endemic JE, all had protective neutralizing antibodies 28 days after the second dose of JE-VC (*14*,*225*).

#### Duration of Neutralizing Antibodies After JE-VC Primary Series

In the pediatric clinical trial of JE-VC in the Philippines, 6 months after completing the primary series, 134 (88%) of 152 children aged 2 months–2 years (95% CI = 82%–92%) and 224 (95%) of 237 children aged 3–17 years (95% CI = 91%–97%) had protective neutralizing antibodies (*14*,*223*). A subset of 300 children were enrolled in a follow-up study and were randomly assigned to groups that received or did not receive a booster dose (*226*). Among the 150 children who did not receive a booster dose, 90% (134 of 149) were seroprotected 11 months after the second dose of the 2-dose primary series, 89% (130 of 146) at 23 months, and 90% (128 of 142) at 35 months (*14*,*226*).

In the observational study of children from countries without endemic JE, 91% (31 of 34) children had protective neutralizing antibodies 6 months after the second dose of the 2-dose primary series (*14*,*225*). Among a subset enrolled in a follow-up study, seroprotection rates were 89% (17 of 19) at 11 months, 91% (21 of 23) at 23 months, and 89% (17 of 19) at 35 months (*14*).

#### Immunologic Response After a Booster Dose

Among 300 children enrolled in a follow-up to the pediatric immunogenicity study conducted in the Philippines, 150 were randomly assigned to receive an age-appropriate booster dose 11 months after the second dose of the primary series. Among these children, 94% (139 of 148) had a protective neutralizing antibody titer before the booster dose, and 100% were seroprotected 1 month, 12 months, and 24 months after the booster dose (Table 7) (*14*,*226*). The GMT was 53 before the booster dose and increased approximately fortyfold to 2,067 at 1 month after the booster dose. GMTs remained high at 428 at 12 months and 350 at 24 months after the booster dose. Data also were used in a mathematical model to estimate the duration of protection after the booster dose and suggested a median duration of 9 years (range: <5 years to ≥30 years) (*227*).

**TABLE 7:** Seroprotection rates and geometric mean titers among children aged 14 months–17 years in the Philippines before and after a booster dose of inactivated Vero cell culture–derived Japanese encephalitis vaccine administered 11 months after the second dose of a 2-dose primary series

Measure	0 days		1 mo		12 mos		24 mos	
Seroprotection rate*	**No. seroprotected (n = 148)**	**(%)**	**No. seroprotected (n = 148)**	**(%)**	**No. seroprotected (n = 147)**	**(%)**	**No. seroprotected (n = 143)**	**(%)**
	139	(94)	148	(100)	147	(100)	143	(100)
**GMT**	**GMT**	**(95% CI)**	**GMT**	**(95% CI)**	**GMT**	**(95% CI)**	**GMT**	**(95% CI)**
	53	(45–64)	2,067	(1,671–2,556)	428	(335–546)	350	(279–440)

**Source:** Kadlecek V, Borja-Tabora CF, Eder-Lingelbach S, et al. Antibody persistence up to 3 years after primary immunization with inactivated Japanese encephalitis vaccine IXIARO in Philippine children and effect of a booster dose. Pediatr Infect Dis J 2018;37:e233–40.

**Abbreviations:** CI = confidence interval; GMT = geometric mean titer.

* Proportion with 50% plaque reduction neutralization test titer ≥10.

### Local and Systemic Adverse Events with JE-VC

JE-VC was studied in approximately 5,000 adults in prelicensure clinical trials (*15*). Local and systemic adverse events caused by JE-VC were similar to those reported for JE-MB or placebo adjuvant alone (*228*,*229*). Subsequent postlicensure studies included children and older adults, and no safety concerns were identified (*206*,*230*). Since the vaccine’s licensure, approximately 1 million doses have been distributed and no patterns of serious hypersensitivity, neurologic, or other serious adverse events considered to be vaccine related have been identified (*231*,*232*).

Among adults, the most common local reactions after a dose of JE-VC are pain and tenderness, and the most common systemic reactions are headache and myalgia (*14*,*228*). Among children, the most frequently reported local reactions are redness in children aged 2 months to <3 years and pain and tenderness in children aged 3–17 years, and the most commonly reported systemic reaction is fever (*14*,*230*).

#### Adverse Events with JE-VC Compared with Placebo Adjuvant

The pivotal safety study comparing 1,993 adults aged ≥18 years randomly assigned to receive 2 doses of JE-VC and 657 persons assigned to receive 2 doses of placebo adjuvant (phosphate buffered saline with 0.1% aluminum hydroxide) indicated similar reactogenicity and adverse events, including medically attended and serious adverse events (*228*). The most common local reactions after administration of dose 1 or 2 of JE-VC were pain (28% and 18% after doses 1 and 2, respectively) and tenderness (29% and 23%), and the most common systemic reactions were headache (22% and 13%) and myalgia (13% and 6%) (*14*,*228*). Two patients had urticaria (*228*). The first patient had a rash localized on the thighs, which occurred 6 days after the second placebo vaccination. The second patient had generalized urticaria of the face, chest, arms, and abdomen, which occurred 8 days after the second dose of JE-VC and was described as moderate; angioedema was not observed. The patient was treated with cetirizine hydrochloride, and the rash resolved after 3 days (*228*). A total of 17 persons, 12 (0.6%) in the JE-VC group and five (0.8%) in the placebo group, terminated the study prematurely because of adverse events (*228*). In the JE-VC group, two of these events (gastroenteritis and rash) were considered severe, and eight of them (headache [two events], influenza-like illness, allergic dermatitis, injection site pain, nausea, fatigue, and rash) were considered to be at least possibly related to the study treatment. No serious neurologic events were identified.

#### Adverse Events with JE-VC Compared with JE-MB

In the noninferiority immunogenicity trial among adults aged ≥18 years, the overall frequency of adverse events reported after JE-VC vaccination (n = 428 persons) was similar to that reported by those receiving JE-MB (n = 435 persons) (*203*). Severe redness, swelling, tenderness, or pain at the injection site were each reported by ≤1% of JE-VC recipients (Table 8). Reported systemic adverse events after JE-VC vaccination generally were mild; the most commonly reported adverse events in the 7 days after each dose were headache (26%), myalgia (21%), influenza-like illness (13%), and fatigue (13%). One serious adverse event was reported in the JE-VC group; a man aged 50 years had a nonfatal myocardial infarction 3 weeks after the second vaccination. The event was considered by the investigator as unlikely to be related to the study vaccine.

**TABLE 8:** Local and systemic adverse events in adults occurring within 7 days after vaccination with inactivated Vero cell culture–derived Japanese encephalitis vaccine or inactivated mouse brain–derived Japanese encephalitis vaccine*

Adverse events	JE-VC^†^	JE-MB**^§^**
**Severe local adverse events**	No. (%) (n = 421)	No. (%) (n = 427)
Redness	4 (1)	46 (11)
Swelling	3 (1)	23 (5)
Hardness	4 (1)	25 (5)
Any^¶^	9 (2)	59 (14)
**Systemic adverse events**	**No. (%) (n = 428)**	**No. (%) (n = 435)**
Headache	113 (26)	125 (29)
Myalgia	88 (21)	69 (16)
Influenza-like illness	54 (13)	55 (13)
Fatigue	54 (13)	48 (11)

**Source:** Tauber E, Kollaritsch H, Korinek M, et al. Safety and immunogenicity of a Vero-cell-derived, inactivated Japanese encephalitis vaccine: a non-inferiority, phase III, randomised controlled trial. Lancet 2007;370:1847–53.

**Abbreviations:** JE-MB = mouse brain–derived Japanese encephalitis vaccine; JE-VC = Vero cell culture–derived Japanese encephalitis vaccine.

* Analysis includes all participants who entered into the study and received ≥1 dose of vaccine.

^†^ Two doses administered at days 0 and 28, with one dose of placebo at 7 days.

**^§^** Three doses administered at days 0, 7, and 28.

^¶^ p<0.01 calculated using Fisher’s exact test for difference between two vaccines.

#### Pooled Safety Data

A pooled analysis of 6-month safety data from seven prelicensure studies among adults aged ≥18 years included 3,558 persons administered at least 1 dose JE-VC, 435 persons administered at least 1 dose of JE-MB, and 657 placebo adjuvant recipients (*229*). Local injection site reactions within 7 days of dose 1 were reported by 48% of the JE-VC persons, 46% of the JE-MB recipients, and 48% of the placebo adjuvant recipients. However, severe local reactions after dose 1 were more frequent after JE-MB (6%) compared with JE-VC (3%) and placebo adjuvant recipients (2%) (p<0.01). Systemic adverse events were reported with similar frequency among persons who received JE-VC (64%), JE-MB (64%), or placebo (61%). Serious adverse events were reported by 1% of the persons in the JE-VC group. Serious allergic reactions did not occur in any of the study groups. Systemic adverse events were reported by a lower percentage of participants after the second dose compared with the first dose.

#### Adverse Events with JE-VC Administered in an Accelerated Primary Series

In the study of adults administered JE-VC in an accelerated schedule concomitantly with a purified chick embryo cell rabies vaccine (n = 217), JE-VC in the standard schedule concomitantly with rabies vaccine (n = 167), or JE-VC in the standard schedule alone (n = 56), local adverse events were reported in 74%, 75%, and 63% of persons, respectively (*205*). Systemic adverse events were reported by 66%, 60%, and 54% of persons in the three groups, respectively. Overall, rates of local and systemic adverse events were similar when JE-VC was administered in an accelerated or standard schedule.

#### Adverse Events with JE-VC in Adults Aged ≥65 Years

In adults aged ≥65 years vaccinated with a 2-dose primary series of JE-VC, in the 7 days after each dose the most common local reaction was tenderness (26%) and the most common systemic reaction was headache (18%) (*206*). Serious or medically attended adverse events were reported in 38 (19%) of 200 persons by day 42 after the second dose, but none were considered by study investigators to be causally related to vaccination.

#### Adverse Events in Children

In an open-label trial in the Philippines, 195 infants aged 2–11 months were randomly assigned to receive JE-VC (n = 131) or 7-valent pneumococcal conjugate vaccine (n = 64). An additional 1,674 children aged 1–17 years were randomly assigned to receive JE-VC (n = 1,280) or hepatitis A vaccine (n = 394) (*14*,*230*). The incidences of local, systemic, medically attended, and serious adverse events were similar between children who received JE-VC or the comparison vaccines. Adverse events were most frequent in children aged 2–11 months. The most frequently reported local reactions were redness in children aged 2 months to <3 years and pain and tenderness in children aged 3–17 years. Overall, 9% (122 of 1,411) of JE-VC recipients had fever (≥100.4°F [38.0°C]) within 7 days after the first dose, and 6% (84 of 1,405) had fever within 7 days after the second dose (*17*). Rates of fever were higher in children aged <3 years compared with older children. Within 1 month after either dose, four (<1%) recipients had urticaria or hypersensitivity reactions, and five (<1%) had neurologic adverse events, including febrile seizures (n = 3), drooling (n = 1), and dizziness (n = 1); all rates were similar to rates for recipients of the comparison vaccines. Solicited local and systemic adverse events were more frequent after dose 1 than dose 2. In children aged 2–11 months, 46% reported events after dose 1 and 28% after dose 2, and in those aged 1–17 years, 32% and 18% reported events after dose 1 and 2, respectively. Among the 1,411 children who received JE-VC, 23 (2%) reported a serious adverse event within 7 months of the first dose. The most common serious adverse events were pneumonia (n = 6) and febrile seizures (n = 5). Only three serious adverse events were reported within 2 weeks after a dose of JE-VC, including one report each of a febrile convulsion, cellulitis, and gastroenteritis. One death from disseminated intravascular coagulation after suspected bacterial meningitis was reported in a boy aged 12 years at 4 months after receipt of the second dose of JE-VC and was considered unrelated to vaccination by the investigator and the trial’s Data Safety Monitoring Board. No other neurologic or hypersensitivity events were reported as serious adverse events.

Among 48 children aged 1–2 years who were randomly assigned to receive JE-VC in a trial in India, five (10%) cases of injection site tenderness and one (2%) case of fever within 7 days after either dose were reported (*224*). The only unsolicited adverse events were one report each of a skin lesion and a skin rash. No serious adverse events or deaths were reported.

In an observational study of children aged 2 months–17 years from countries without endemic JE, adverse events were evaluated among 12 children who received a 0.25-mL dose and 88 children who received a 0.5-mL dose (*14*,*225*). Among children who received the 0.25-mL dose, the most common solicited adverse reactions within 7 days after either JE-VC dose were injection-site redness (n = 3, 25%) and diarrhea (n = 2, 17%). Among children who received the 0.5-mL dose, the most common solicited adverse reactions were injection-site tenderness (n = 44, 50%) and muscle pain (n = 27, 31%). Three serious adverse events were reported. None occurred within 28 days of either dose of JE-VC. One child each had diabetes mellitus (3 months after dose 2), dizziness (4 months after dose 2), and intentional self-injury.

#### Adverse Events After a JE-VC Booster Dose in Adults and Children

Among adults aged ≥18 years who received a JE-VC booster dose at 15 months after the first dose of a 2-dose primary series, during the 7 days after the booster dose the most frequent local reactions were tenderness in 19% (37 of 193) and pain in 13% (25 of 195), and the most commonly reported systemic reactions were headache in 11% (21 of 194) and fatigue in 10% (18 of 188) (*16*,*215*,*233*). No serious adverse events were reported during the 28 days after the booster dose.

In a similar trial conducted among children, within the 7 days after the booster dose, 12 (8%) of 148 children had a local adverse event, and 21 (14%) had a systemic adverse event (*226*). Two children experienced serious adverse events within 1 month after the booster dose. One child had an abscess in the lumbar area 1 day after vaccination, and one had dengue fever approximately 4 weeks after the booster dose.

#### Adverse Events with Concomitant Administration of JE-VC and Hepatitis A or Rabies Vaccines

Persons in a clinical trial who received the first dose of JE-VC administered concomitantly with hepatitis A vaccine were more likely to report pain, redness, and swelling than persons who received either vaccine alone (*209*). No other differences were reported in safety or reactogenicity with concomitant administration of JE-VC and hepatitis A vaccine compared with administration of each vaccine alone.

Adults receiving JE-VC and rabies vaccine reported more local and systemic adverse events when the vaccines were coadministered compared with administered alone (*205*,*220*). Among 166 persons who received the vaccines concomitantly, local reactions were reported in 125 (75%) and systemic reactions in 100 (60%). Among 56 persons who received JE-VC alone, 35 (63%) reported local reactions and 30 (54%) reported systemic reactions.

#### Postlicensure JE-VC Surveillance

Two reviews of vaccine safety data from the U.S. Vaccine Adverse Event Reporting System (VAERS) have occurred since vaccine licensure, covering a total of 7 years during May 2009–April 2016, when >1 million doses of JE-VC were distributed (*231*,*232*). VAERS is the national passive surveillance system for monitoring adverse events after vaccination with reports submitted by health care providers, vaccine recipients, and vaccine manufacturers. The overall rates of adverse events in the two analyses were similar, with a rate of 15.2 adverse events per 100,000 doses distributed in the first analysis and 14.8 adverse events per 100,000 doses during the second period. The rates were similar to or lower than the 15.0 and 23.7 adverse events per 100,000 doses distributed previously reported to VAERS for JE-MB (*234*,*235*). In the two analyses of JE-VC data reported to VAERS, the rates of serious adverse events defined according to the FDA definition were 1.8 and 1.1 per 100,000 doses distributed; among the 14 serious event reports, 11 (79%) were reports in which JE-VC was administered with one or more other vaccines (*236*). Hypersensitivity events were reported at rates of 3.0 and 4.4 per 100,000 doses distributed, and 56% (20 of 36) occurred after concomitant administration of JE-VC with other vaccines. Neurologic events were reported at rates of 2.2 and 1.2 events per 100,000 doses distributed. The neurologic adverse event reports included four reports of seizures after vaccination, and all occurred after administration of JE-VC with other vaccines. VAERS data cannot generally be used to determine causality, especially among persons who receive multiple vaccines. However, the majority of reports in both analyses were not serious, and no unexpectedly high reporting rates for specific events were identified.

In a postmarketing adverse event surveillance study conducted among U.S. military personnel, rates of hypersensitivity and neurologic reactions were much higher, reflecting the different study methods (*237*). An active surveillance approach was used, events were identified using *International Classification of Diseases, Ninth Revision,* codes, and a retrospective review of medical records was conducted. However, complete descriptions of events often were lacking, preventing clarification of the nature of some events. In addition, the assessment was conducted among military personnel who sometimes received multiple other vaccines with JE-VC, including reactogenic vaccines.

### Vaccination of Pregnant or Breastfeeding Women 

No controlled studies have assessed the safety, immunogenicity, or efficacy of JE-VC in pregnant women. Preclinical studies of JE-VC in pregnant rats did not show evidence of harm to the fetus (*14*). No studies have investigated the safety or immunogenicity of JE-VC in breastfeeding women, and no data are available on whether JE-VC is excreted in human milk. ACIP general guidelines for best vaccination practices indicate inactivated vaccines administered to breastfeeding women do not affect the safety of breastfeeding for women or their infants (*238*).

### Cost-Effectiveness of JE Vaccines

Several studies have demonstrated that JE vaccination among children in JE-endemic countries is cost-effective or cost-saving compared with no vaccination (*239*–*241*). Because of the substantially lower risk for disease among U.S. travelers and use of a much higher cost vaccine than those used for routine vaccination programs in Asia, JE vaccination for travelers would not be expected to be cost-effective. However, cost-effectiveness is less relevant for travel vaccines that usually are paid for by the travelers themselves and are not covered by most insurance plans or the Vaccines for Children program.

One comparative analysis compared strategies for JE vaccination for U.S. travelers to Asia among three groups (*242*). Group l included higher-risk travelers who planned to spend ≥1 month in JE-endemic areas, group 2 included travelers who would spend <1 month in JE-endemic areas with at least 20% of their time participating in outdoor activities in rural areas, and group 3 included the remainder of shorter-term and lower-risk U.S. travelers to Asia. An analytic horizon of 6 years was used, although productivity losses were evaluated over average life expectancy. To prevent one JE case, the number of travelers who would need to be vaccinated was 0.7 million, 1.6 million, and 9.8 million in groups 1, 2, and 3, respectively. The cost to prevent one JE case from a societal perspective was approximately $0.6 billion, $1.3 billion, and $7.9 billion for each group. The variable with the greatest influence on the cost-effectiveness of vaccination was disease incidence among travelers, and a sensitivity analysis was conducted increasing baseline incidence 100 times. Using this higher incidence, in groups 1, 2, and 3 the numbers of travelers needed to be vaccinated to prevent a case were 7,000, 16,000, and 98,000, and the cost per case averted was $5 million, $12 million, and $78 million, respectively. Although the cost per case averted was high for all groups of travelers, this comparative analysis supported focusing on vaccination of travelers at increased risk for disease compared with those at lower risk.

## Recommendations for the Prevention of JE Among U.S. Travelers

JE is a very low-risk disease for most U.S. travelers to JE-endemic countries. However, some travelers are at increased risk for infection on the basis of their planned itinerary. Factors that increase the risk for JE virus exposure include 1) longer duration of travel; 2) travel during the JE virus transmission season; 3) spending time in rural areas; 4) participating in extensive outdoor activities; and 5) staying in accommodations without air conditioning, screens, or bed nets (Box 2).

BOX 2 **Factors that increase risk for Japanese encephalitis among travelers**

**Duration**

- Highest incidence of disease has been reported among longer-term travelers.
- Although no specific duration of travel puts a traveler at risk for JE, longer-term travel increases the likelihood that a traveler might be exposed to an infected mosquito.
- Longer-term travel includes cumulative periods in JE-endemic areas; this includes frequent travelers and persons living in urban areas who are likely to visit higher-risk rural areas.

**Season**

- JE virus transmission occurs seasonally in some areas and year-round in others.
- Information on expected JE virus transmission by country is available from the *Yellow Book* on the CDC website (https://wwwnc.cdc.gov/travel/yellowbook/2018/infectious-diseases-related-to-travel/japanese-encephalitis). These data should be interpreted cautiously because JE virus transmission varies within countries and from year to year.

**Location**

- The highest risk occurs from mosquito exposure in rural or agricultural areas.
- Mosquitoes that transmit JE virus typically breed in flooded rice fields, marshes, and other stagnant collections of water.
- Some cases have been reported among travelers to coastal areas or resorts located in or adjacent to rural or rice growing areas.
- JE can occur in large, focal outbreaks indicating extensive active JE virus transmission in those areas.

**Activities**

- The mosquitoes that transmit JE virus feed most often in the outdoors, particularly from sunset through dawn; therefore, examples of activities that increase risk include the following:
  - Outdoor recreational activities such as camping, hiking, trekking, biking, rafting, fishing, hunting, or farming.
  - Spending substantial time outdoors, especially during the evening or night.

**Accommodations**

- Accommodations without air conditioning, screens, or bed nets increase risk for mosquito exposure.

**FIGURE 3:**
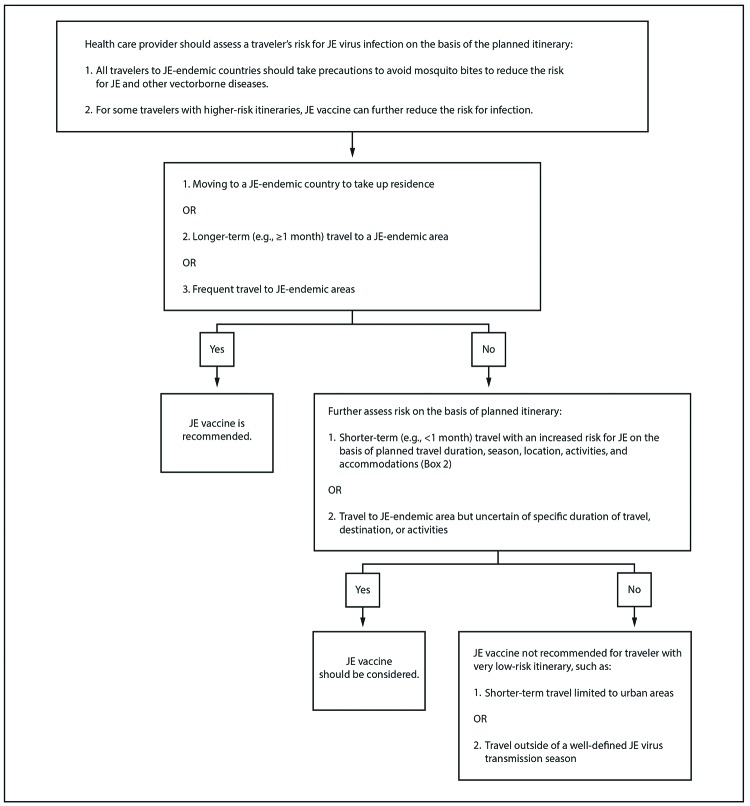
Vaccine recommendations for U.S. travelers to areas with endemic Japanese encephalitis **Abbreviation:** JE = Japanese encephalitis.

Health care providers should assess each traveler’s risk for mosquito exposure and JE virus infection on the basis of their planned itinerary and discuss ways to reduce their risk (Figure 3). All travelers to JE-endemic countries should be advised to take precautions to avoid mosquito bites to reduce the risk for JE and other vectorborne diseases. These precautions include using insect repellent, permethrin-impregnated clothing, and bed nets and staying in accommodations with screened or air-conditioned rooms.

For some persons who might be at increased risk for JE based on travel duration, season, location, activities, and accommodations, JE vaccine can further reduce the risk for infection. The decision whether to vaccinate should be individualized and consider the 1) risks related to the specific travel itinerary, 2) likelihood of future travel to JE-endemic countries, 3) high morbidity and mortality of JE, 4) availability of an effective vaccine, 5) possibility but low probability of serious adverse events after vaccination, and 6) traveler’s personal perception and tolerance of risk.

JE vaccine is recommended for persons moving to a JE-endemic country to take up residence, longer-term (e.g., ≥1 month) travelers to JE-endemic areas, and frequent travelers to JE-endemic areas. JE vaccine also should be considered for shorter-term (e.g., <1 month) travelers with an increased risk for JE based on planned travel duration, season, location, activities, and accommodations (Box 2). Vaccination also should be considered for travelers to JE-endemic areas who are uncertain of specific duration of travel, destinations, or activities. JE vaccine is not recommended for travelers with very low-risk itineraries, such as shorter-term travel limited to urban areas or travel that occurs outside of a well-defined JE virus transmission season.

## Recommendations for the Prevention of JE Among Laboratory Workers

Work with JE virus is primarily restricted to biosafety level 3 (BSL-3) facilities and practices; however, the attenuated SA14-14-2 JE vaccine virus can be handled at BSL-2 (*243*). In a laboratory setting, JE virus might be transmitted through accidental percutaneous, or theoretically, mucosal or inhalational exposures. Vaccine-induced immunity presumably protects against exposure through a percutaneous route. Exposure to aerosolized JE virus, particularly high concentrations that might occur during viral purification, might lead to infection through mucous membranes or through the olfactory epithelium directly into the central nervous system. Whether vaccination provides protection after such exposures is unknown.

Vaccination is recommended for all laboratory workers with a potential for exposure to JE viruses other than SA14-14-2 JE vaccine virus. Vaccination generally is not required for those who work only with SA14-14-2 JE virus; however, for those working with SA14-14-2 virus at high concentrations or volumes, or passaging virus, individual risk assessments with consideration of biosafety level and vaccination should be undertaken by a local biosafety committee. Vaccination is not required for workers handling routine clinical samples.

## Administration of JE Vaccine

### Vaccine Composition, Presentation, and Storage

Each 0.5-mL dose of JE-VC contains 250 *μ*g aluminum hydroxide as an adjuvant. The finished product does not include gelatin stabilizers, antibiotics, or thimerosal. JE-VC is supplied in a 0.5-mL prefilled glass syringe with a plunger stopper (chlorobutyl elastomer, with no natural latex rubber). The vaccine should be stored at 35°F–46°F (2°C–8°C) and should not be frozen. The vaccine should be protected from light.

### Dosage, Schedule, and Administration

#### Primary Vaccination Series

The vaccination dose and primary schedule for JE-VC vary by age (Box 1).

- **2–35 months:** 2 doses (0.25 mL each) administered intramuscularly (IM) on days 0 and 28.
- **3‒17 years:** 2 doses (0.5 mL each) administered IM on days 0 and 28.
- **18‒65 years:** 2 doses (0.5 mL each) administered IM on days 0 and 7‒28; this is the only age group for which an accelerated schedule is approved.
- **>65 years:** 2 doses (0.5 mL each) administered IM on days 0 and 28.

For all age groups, the 2-dose series should be completed at least 1 week before potential exposure to JE virus.

#### Booster Dose

For adults and children, a booster dose (i.e., third dose) should be given ≥1 year after completion of the primary JE-VC series if ongoing exposure or reexposure to JE virus is expected. The booster dose for children aged <3 years is 0.25 mL and for adults and children aged ≥3 years is 0.5 mL. No data are available on the response to a booster dose administered >2 years after the primary series. Clinical trial data show high rates of seroprotection for at least 6 years after a booster dose (*217*); no longer-term study data are available. No U.S. recommendations exist on the need for subsequent booster doses.

#### Vaccine Preparation

During storage, the vaccine might appear as a clear liquid with a white precipitate. Before administration, shake the syringe well to obtain a white, opaque, homogeneous suspension. To administer a 0.25-mL dose, expel and discard half of the volume from the 0.5-mL prefilled syringe by pushing the plunger stopper up to the edge of the red line on the syringe barrel before injection. See the prescribing information for additional information on preparing the 0.25-mL dose (*14*).

#### Simultaneous Administration of Other Vaccines or Drugs

A clinical trial in which the first dose of JE-VC was administered concomitantly with hepatitis A vaccine indicated no interference with the immune response to JE-VC or hepatitis A vaccine (*209*). Similarly, noninferiority of the immunological responses to JE-VC and purified chick embryo cell culture rabies vaccine was established for concomitant administration of the two vaccines compared with separate administration of either vaccine (*205*,*220*). If JE-VC and other vaccines are administered concomitantly, they should be administered with separate syringes and at different anatomical sites (i.e., >1 inch apart if possible).

## Contraindications and Precaution for the Use of JE Vaccine

### Allergy to Vaccine Components

A severe allergic reaction (e.g., anaphylaxis) after a previous dose of JE-VC, any other JE vaccine, or any component of JE-VC is a contraindication to administration of a subsequent dose. JE-VC contains protamine sulfate, a compound known to cause hypersensitivity reactions in some persons (*14*).

### Pregnancy

Pregnancy is a precaution for the use of JE-VC. Vaccination with JE vaccine usually should be deferred because of a theoretical risk for the developing fetus. However, pregnant women who must travel to an area in which risk for JE is high should be vaccinated if the benefits outweigh the risks of vaccination to the mother and developing fetus.

## Special Populations

**Infants aged <2 months:** Safety and effectiveness of JE-VC have not been established for infants aged <2 months.
**Adults aged ≥65 years:** In a postlicensure observational study conducted among older adults, both the seroprotection rate and GMT were substantially lower after the primary JE-VC series compared with rates in younger persons. However, no data are available on the safety or immunogenicity of an additional dose or early booster dose of JE-VC for adults aged ≥65 years.
**Breastfeeding women:** Breastfeeding is not a contraindication or precaution to vaccination with JE-VC.
**Persons with altered immune states:** No data exist on the use of JE-VC in immunocompromised persons or patients receiving immunosuppressive therapies; however, these persons might have a diminished response to JE-VC.

## Reporting of Vaccine Adverse Events

Surveillance for adverse events associated with administration of JE vaccine is important. Even if a causal relation to vaccination is not certain, all clinically significant adverse events should be reported to the VAERS (https://vaers.hhs.gov or 800-822-7967).

## Future Research on JE-VC

Additional studies of JE-VC would be useful to evaluate persistence of protective immunity beyond 6 years after a booster dose, response to a booster dose administered >2 years after the primary JE-VC series, and response to a booster dose in adults aged >65 years.

## Additional Information

Additional information about JE is available from CDC at https://www.cdc.gov/japaneseencephalitis and in the CDC *Yellow Book* (*4*). Additional licensure information for JE-VC is available from the U.S. Food and Drug Administration (https://www.fda.gov/vaccines-blood-biologics/vaccines/ixiaro).
